# A Critical Review of Horse-Related Risk: A Research Agenda for Safer Mounts, Riders and Equestrian Cultures

**DOI:** 10.3390/ani5030372

**Published:** 2015-07-17

**Authors:** Kirrilly Thompson, Paul McGreevy, Phil McManus

**Affiliations:** 1 The Appleton Institute, Central Queensland University, 44 Greenhill Road, Wayville, SA 5034, Australia; 2 Faculty of Veterinary Science, University of Sydney, Sydney, NSW 2006, Australia; E-Mail: paul.mcgreevy@sydney.edu.au; 3 School of Geosciences, University of Sydney, Sydney, NSW 2006, Australia; E-Mail: phil.mcmanus@sydney.edu.au

**Keywords:** horse-riding, risk, mitigation, culture, research, review, safety, behavior change, eque-culture, motivator

## Abstract

While the importance of improving horse-related safety seems self-evident, no comprehensive study into understanding or reducing horse-related risk has been undertaken. In this paper, we discuss four dimensions of horse-related risk: the risk itself, the horse, the rider and the culture in which equestrian activities takes place. We identify how the ways in which risk is constructed in each dimension affects the applicability of four basic risk management options of avoidance, transference, mitigation and acceptance. We find the acceptance and avoidance of horse-related risk is generally high, most likely due to a common construction of horses as irrevocably unpredictable, fearful and dangerous. The transference of risk management is also high, especially in the use of protective technologies such as helmets. Of concern, the strategy least utilised is risk mitigation. We highlight the potential benefit in developing mitigation strategies directed at: (a) improving the predictability of horses (to and by humans), and (b) improving riders’ competence in the physical skills that make them more resilient to injury and falls. We conclude with the presentation of a multidisciplinary agenda for research that could reduce accident, injury and death to horse-riders around the world.

## 1. Background

Horse-riding is a hazardous activity. Each year, horse riders are injured, hospitalized or killed as a result of horse-related accidents and injuries. Despite technological advancements in equestrian safety equipment [1], horse riding continues to be found more dangerous than motorcycling, skiing, football, and rugby [2,3]. Whilst injury can occur simply from handling horses [4,5,6,7], falling from a horse constitutes a dangerous fall from height, possibly at speed. A rider’s head can be elevated up to 3 m from the ground and horses can travel at speeds around 50 km/h. one study of children under 15 years of age found that a ‘mean Modified Injury Severity Scale score of injured riders was exceeded only by that of pedestrians struck by a car’ [8] (p. 487).

Improving safety for the millions of horse-riders around the globe is significant for moral, economic, socio-cultural, and public health reasons. While the importance of improving horse-related safety seems self-evident, no rigorous study into understanding or reducing horse-related risk has been undertaken internationally in the academic literature. This may be due to a historical legacy of horse-riding being a pre-modern sport with a robust culture that accepts the dangers of riding. It may also be due to the difficulty of analyzing and mitigating the risks inherent in, and generated by, a complex socio-technical network and interspecies interaction that is historically, socially and culturally constructed. Nonetheless, complexity must be addressed to enhance the safety of millions of equestrians around the globe.

In this paper, we discuss four inter-related dimensions of horse-related risk: the risk itself, the horse, the rider and the cultural context through which equestrian activities become meaningful. For each dimension, we present an overview of current knowledge and offer a list of important questions that remain. Moreover, we identify the repercussions of how risk has been constructed for each dimension. Risk management strategies are commonly presented as four options: avoidance, transference (of responsibility to a third party), mitigation and acceptance [9]. As argued throughout this paper, the acceptance and avoidance of horse-related risk is high. Horses are frequently constructed as irrevocably unpredictable and dangerous. As a result, most horse-related safety information concentrates on avoiding injury by keeping out of horse-related danger. Risk transference is also high, in relation to a reliance on insurance companies, professional trainers to manage difficult horses or the use of protective technologies such as helmets, body protectors or even sedative substances. Lowest of all is mitigation, particularly mitigation directed at: (a) improving the predictability of horses (through making horses more predictable and making riders more able to predict their behavior); and (b) improving riders’ competence in the physical skills with the explicit intention of making them more resilient to injury and falls.

To address the questions raised throughout the paper, we conclude with a list of recommended methods for future data collection. Together, these questions and methods comprise a multidisciplinary agenda for research that could reduce accident, injury and death to millions of horse-riders around the world.

## 2. Current Knowledge and Critical Questions

### 2.1. The Risk

As noted above, horses are dangerous and interacting with them is more or less risky. Horses are often pushed to their physical and physiological limits in equestrian pursuits [10], resulting in risk to both horses and riders. Research on horse-related risk is biased towards cross-sectional epidemiological studies of injury type and severity [7], or the efficacy of technical interventions such as frangible pins on jumping obstacles, helmets and back protectors [8,11,12,13]. With very little exception [13,14,15], a focus on the causes and consequences of horse-related injury rather than prevention, could lead to researchers being accused of ‘shutting the gate after the horse has bolted’.

Whilst risk analysis is an important aspect of risk research, especially for ranking risk, prioritizing intervention and evaluating campaigns, risk-reduction requires changing the attitudes and behaviours of participants in risky activities. Sandman’s model of risk communication is particularly useful in this regard [16]. According to Sandman, ‘risk’ is most usefully understood not as the standard arithmetical product of likelihood and consequence of an incident occurring (ISO 31000), but as the cultural sum of ‘hazard + outrage’. Sandman discusses combinations of high/low risk/outrage on a 2X2 matrix and their implications for crisis management. For example, the risk of autism being induced by childhood immunization is a ‘low risk/high outrage’ concern. The cultural response of high outrage outweighs and overshadows the objective risk but has had a significant impact on rates of failure to immunize.

Horse-related risk can be considered ‘high risk/low outrage’, suggesting a level of wide-spread recognition of risk but a high degree of acceptance, complacency or inaction. The recommended strategy for encouraging behaviours that reduce horse-related risk would then be to reduce the risks (see sections on the rider and the horse below), whilst also increasing outrage (see ‘culture’, below). In particular, outrage over horse-related injury needs to be increased amongst riders as well as the governments that have the ability to financially support or legislate for change. As increasing outrage when there is no available risk mitigation can lead to paralyzing fear and perpetuate complacency, there is a need to fortify existing technical risk controls with behavioural, physical and cultural controls.

In relation to the risk dimension of horse-related risk, we therefore need to ask: How can horse-related injuries be prevented?

(a) What contributes to complacency around horse-related risk?
(b) How can outrage over horse-related risk be usefully increased?
(c) What behavioural, physical and cultural controls can supplement existing technical controls?

### 2.2. The Horse

The risk of horse-related accidents and injury are well known and widely accepted. Risk is frequently attributed to the ‘nature’ of horses as irrevocably unpredictable, fearful and flight-wired [17]. As noted by Lawrence, even the most highly trained horses can be treated with suspicion, lest they go ‘feral’ and return to a state of unruly wildness [18]. This essentialist view of horses as more or less ‘unchangeable’ could explain why horse-related risk intervention is largely technical, and risk management is avoidance-based (as discussed in more detail below).

In equestrianism, there has been a strong focus on using personal protective equipment (e.g., helmet, boots, gloves and, more recently, body protectors) and maintaining horse equipment to safe standards (e.g., checking stitching on saddlery). Whilst technical intervention has an undeniable role in reducing the likelihood and consequence of an adverse equestrian event, the voluntary use of basic protective equipment such as helmets use is low to inconsistent [11,12,15]. Technical intervention should therefore not overshadow attention to horse behavior, or rider skill (discussed in more detail below).

Overall, there seems to have been more discussion about why horses are dangerous (e.g., unpredictable, large and flighty), rather than if, how and to what extent those risk factors can be mitigated or even controlled. In fact, research suggests that the unpredictability of horses can be reduced through behavioural interventions and approaches [19]. This is particularly encouraging, given that a taken-for-granted assumption that horses are irrevocably unpredictable may contribute to a lack of outrage about current levels of horse-related accident, injury and death. Even if horses are irrevocably unpredictable based on the innate fears or a prey animal, there has been no consideration of the extent to which their own need to feel safe could be reconfigured to reduce risk to humans. As noted by McGreevy and colleagues, ‘the value of safety to animals is often overlooked by trainers and handlers’ [20]. This is a firm reminder that feelings of risk, fear and safety are at least as important to horses as well as people. As these equine desires may contribute to risks where their importance goes unrecognized, the corollary is that they could be used to reduce risk where their importance is accommodated and addressed.

The unpredictability of horses suggests that risks may be higher amongst people with low levels of knowledge of horse ethology and behaviour, since this can help to anticipate natural but undesirable equine behaviours and responses. Evidence of breed differences in personality [21] and vision [22] has been revealed, but there is little guidance on how to use this information to reduce the risks associated with handling and riding certain horses. One exception is the recent ‘Guide to managing risks when new and inexperienced persons interact with horses’ [23], that singles out Thoroughbred horses as unsuitable for beginners. This seminal guide highlights the potential risk reduction benefits of applying WHS principles to equestrian cultures. In workplaces, workers are given guidance and decision-making support tools to safely operate dangerous plant, machinery or equipment through the identification and control of hazards. However, professional, amateur or leisure riders are rarely given decision-making tools for riding a given horse on a given occasion (*i.e.*, the equivalent of a pre-flight safety check). Although evidence of its adoption is lacking, the following seven point pre-ride equestrian checklist has been suggested by Guyton *et al*. [24]:

(1) Am I wearing adequate protective gear?
(2) Is the tack durable and well-fit to the horse?
(3) Are the environmental conditions (weather, ground footing) safe for riding?
(4) Does the arena contain unfamiliar objects, animals, or people that may alarm the horse?
(5) Is the horse healthy and prepared for riding? Is the mood or behavior of the horse uncharacteristic or concerning?
(6) Am I healthy and prepared for riding? Do I have an emotional or physical condition that may impair my ability to safely ride this horse?
(7) Do the horse and I have a healthy relationship? Do I have concerns about my ability to assert myself with this horse? [24]

The list is entirely relevant, but can be significantly improved. The first two checks relate to technology and are under direct control of the human. Checks Three and Four relate to the environment, over which riders have less control, especially when riding in open spaces. Check Six relates to the rider but ‘emotional or physical’ condition is rather broad, especially around the degree to which safety is jeopardized. Checks Five and Seven relate to the horse, but require familiarity with individual horses or a perceptive eye informed by knowledge of equine behavior. The idea of a ‘healthy relationship’ and being ‘assertive’ are both open to interpretation and relative to different equestrian disciplines. Moreover, being able to interpret and predict equine behaviour may be a more useful risk-management tool than being assertive. Check Six relates to the rider, but ‘emotional or physical’ condition is rather broad, especially around the degree to which safety is jeopardized by particular conditions. Furthermore, Check Six does not address specific physical skills essential for riding, such as those discussed in more detail below.

Overall, whilst the checklist comprises questions that are undoubtedly important to ask, most if not all rely on a level of knowledge and expertise in their interpretation and assessment. There is particular scope to incorporate objective checks of safety, quality of the human-horse relationship and resilience to external or environmental stimuli. These can be undertaken first from the ground and then under saddle in a confined area through testing basic responses such as deceleration, acceleration (sometimes necessary to avoid hazards such as vehicles or other horses), reversing and turning.

Moreover, riders are not routinely given explicit advice on what to do if they assess a horse as unsafe, *i.e.*, additional controls, alternative (less dangerous) activities, strategies to improve the safety of a mount (*i.e*., install robust deceleration responses and habituate the horses to common hazards). Whilst riders may be given instruction that has the effect of improving safety, it may not be understood or framed explicitly as a risk-management strategy. Rather, it may be seen as taken for granted tradition or simply ‘just the way things are done’, as has been found in relation to risk management by volunteer firefighters [25]. Riders too may already be engaging in risk-reduction practices that are tacit. The explicit identification of practices that improve safety as risk-reduction strategies may encourage riders not only to value, improve and maintain high standards of those practices and the horse’s response to them, but to communicate those practices to other riders to create safer equestrian cultures.

It is tempting to construct the horse as an independent source of risk. Certainly, without a horse there is no risk. However, from a safety systems perspective, there is a need to recognize the ways in which risk is generated in, by and through socio-technical networks. Despite the importance of matching riders with appropriate horses being acknowledged [24] and largely carried out on a basis of experienced/inexperienced horse/rider [26], there is no widely accepted or validated means of assessing experience for the purpose of determining safe horse-rider combinations. As environmental conditions and stressors can impact the behavior of horses and riders, assessment needs to be undertaken in general as well as on specific occasions, such as during competition, on return to work after an injury or in unfamiliar surroundings.

In relation to the equine dimension of horse-related risk, we therefore need to ask:

(a) How well do riders understand horse behavior?
(b) How can increasing levels of knowledge about horse behavior improve safety?
(c) Which horses are most likely to be unpredictable for riders (e.g., age, breed, level of experience, level of horse education, early preparation and history of unwelcome behaviour)
(d) What physical and behavioural conditioning increases a horse’s predictability or make a horse reasonably safe to ride?
(e) How might a horse’s preference for ‘safety’ be used to reduce risk?
(f) What benefits may be taken from OHS approaches to safety?
(g) How can a rider assess, train, maintain and improve a horse’s level of risk?
  - From the ground
  - From the saddle
(h) What tacit practices do riders currently engage in without being aware of their risk-reduction benefits, which could be emphasized?
(i) What tools could enable safer matching of horse and rider?

### 2.3. The Rider

Most—if not all—riders are aware of the risk of equestrian sports and mindful of their own safety [27]. Pony club manuals are replete with references to safe practices around horses [28,29,30] and the pony club movement itself has a ‘safety first’ attitude. However, from a risk management perspective, the most common strategies for improving safety around horses can be characterized as avoidance strategies—keeping out of harm’s way. Aside from the safe use of safe equipment, being ‘horse safe’ usually refers to how to avoid being kicked, bitten, trampled or crushed. Once again, the reliance on avoidance rather than control could be a repercussion of the construction of horses as irrevocably unpredictable. As discussed above, there is very little overt discussion about how to manage or reduce ‘equinogenic’ risk.

Similarly, there is little overt discussion of the physical and postural skills that can increase a rider’s resilience to injury or falls. Riders communicate to horses through ‘natural aids’; their hands (rein tension increases for ‘stop’ and ‘turn’), legs (leg pressure increases for ‘go’), seat (classically conditioned weight and balance cues for ‘stop’ or ‘go’), voice and various ‘artificial aids’ such as whips and spurs. The self-awareness, timing and accuracy of the application of aids have been related to rider safety, especially (1) rein pressure [tension and release], (2) leg stability and (3) balance and trunk stability [31]. When these stimuli are applied incorrectly, inadvertently or simultaneously, they can cause miscommunication, confusion, discomfort or pain for the horse. The horse may respond with evasive behaviour, resistance or flight behaviour and anti-predator responses such as rearing, bucking or bolting, potentially leading to rider injury or even death. Riders’ cues must be applied with awareness, accuracy, timing and consistency to: (a) avoid or reduce the likelihood of a horse-related incident occurring; and (b) provide the rider with the ability to safely respond to an untoward incident, thereby reducing the significance of its consequences [32].

The mensuration of cues from riders’ hands and legs has commenced as part of equitation science [33]. There may also be benefits in measuring levels of cognitive and somatic anxiety in riders, as anxiety can impact horse-rider communication by affecting fine motor control and decision-making [34,35]. High self-efficacy may reduce anxiety [36], although little is known about riders’ subjective confidence in their proficiency in the application of those cues, based on self-report. From a psychological perspective, high self-efficacy may mislead riders into over-estimating their safety and/or taking increased risks [37,38].

Furthermore, there is a need to know if and how riders associate their physical skills with safety, especially in some of the more artistic equestrian disciplines such as dressage where a rider’s skills have significant aesthetic value. There are several well established approaches to improving riding position such as ‘centred riding’ [39] and ‘riding with your mind’ [40] that focus on riders developing better feel, relating better with the horse, and becoming more effective as well as feeling and looking more aesthetically pleasing. The simultaneous effect of these approaches on improving safety is largely taken for granted and remains to be determined, as does the usefulness of ‘improved safety’ as a means to motivate riders to adopt such approaches.

In relation to the rider dimension of horse-related risk, we therefore need to ask:

(a) How riders acquire and objectively perform skills related to horse safety?
(b) How riders subjectively self-evaluate the skills essential to horse safety?
(c) If and how riders and trainers associate physical ability with safety?
(d) What is the impact of existing rider position programmes on safety?

### 2.4. The Culture

As acknowledged by Sandman’s hazard/outrage model descried above, subjective perceptions of risk do not always align with objective risk calculations [41], and not all strategies that reduce fear actually reduce risk. Perceptions of risk can have a significant impact on the uptake of safety behaviours and protective equipment. ‘Safety culture’ is a contested term that has been used in studies of organizational culture to describe the ways in which workplaces do (or do not) value safety [42,43] or perform on measures thought to indicate an organisation’s ‘safety climate’ [44]. The concept can be used to consider the ways in which various equestrian cultures or ‘eque-cultures’ [45] impact on the safety of equestrians, through cultural variation in risk perceptions, attitudes, beliefs and behaviours. In relation eque-culture in general, safety is often poorly evaluated especially at the point of sale [14,46]. This is despite a growing appreciation of the horse’s need to feel safe [20] and the range of responses a horse offers when threatened [47]. In relation to racing eque-cultures, the common rehoming of racehorses to novice riders is one example of a contemporary eque-cultural practice that presents risks to horses and humans. Whilst the aim is often to reduce the risk of horses being unnecessarily euthanized, this practice may increase the risk of a horse-related risk or injury to a human, and the potential mistreatment of a horse that is subsequently labeled ‘dangerous’.

From a more socio-historical standpoint, the romantic cultural construction of horse riding as an art can also conflict with practical considerations of risk. Since the Classical Greek period and especially in the renaissance in Europe, equitation has been constructed as an art [48,49,50]. As with any artistic endeavor, there is reverence for those who seem blessed with a natural affinity or ‘feel’ that eludes explanation. In equestrian culture, there has always been an acceptance that some equestrians have a natural ability to communicate seemingly telepathically with horses such that the two become one [51,52]. These riders are said to have natural ‘feel’ [26]. However, equitation science is helping to demystify many of the qualities that distinguish such individuals [32,53,54,55]. For example, communication from rider to horse always relies upon at least some form of pressure cues, however subtle [55]. These cues appear imperceptible in the best cases having been reduced from larger operant cues [19] by a process of classical conditioning [56]. Different eque-cultures have different tolerances for the ‘volume’ of cues to the horse and their visibility to bystanders.

Whilst equitation science has the potential to significantly reduce horse-related injury and death, it would be disingenuous to present it as a panacea, at least uncritically. In any given population, there will be varying levels of scientific literacy and diverse attitudes towards ‘scientific’ modes of thought, ranging from supporters to skeptics. For some riders, the elusive experience of harmony is precisely what attracts them to horse-riding [51]. For others, instruction from elite competitors is highly valued [26,27]. Some resist a scientific framework of horse training, preferring leadership narratives that place them above their horses in a perceived hierarchy [57]. Moreover, research has identified that the desire to achieve a ‘good’ human-horse relationship (or at least to ‘perform’ the achievement of a ‘good’ relationship) can be a barrier to precautionary behaviour when such riders consider their ‘good’ relationship as evidence of reduced risk and therefore a reduced need to wear protective equipment such as helmets [15].

This heterogeneity of styles of engagement with equestrianism, or ‘equestrian dispositions’, suggests that safety information needs to be communicated in forms that resonate with riders of a multitude of dispositions including scientific and artistic approaches to equitation. Understanding the cultural specificity and generalizability of eque-cultures and equestrianism more broadly will be crucial to effectively applying or adapting longstanding behavior change theories and models from health psychology [58,59,60,61]; as well as theories of decision-making and threat and error management from human factors and safety science [62]. Whilst different equestrian dispositions have been operationalized from researcher experience [57] and through factor analysis [63], there remains a need to evaluate their resonance with equestrians themselves. There is a particular need to understand what motivates the behaviour of different equestrians to identify useful ‘irrelevant motivators’ [64] that could encourage the adoption of protective behaviours in the short term and cultivate safety values in the medium to long term. For example, signaling being fashionable or professional may be a powerful motivator for some riders to wear helmets [65]. Others might be more motivated to wear helmets if they become a symbol of the ‘toughness’ of their discipline, especially in traditional cowboy equestrian disciplines associated with masculinity, bravery and resilience [66]. Regardless of the motivation, many riders will be influenced by their peers, and could be engaged to replicate desired precautionary behaviours through ‘participant modeling’ [36] by their role models.

In relation to the cultural dimension of horse-related risk, we therefore need to ask:

(a) How do riders perceive equestrianism in general and equitation science in particular?
(b) What are the different styles of engagement with equestrianism?
(c) What are the most powerful motivators for equestrians?
(d) How do riders consume safety-related information?
(e) How are the causes of horse-related injuries and deaths understood by riders?
(f) How is the use of horse safety equipment represented in the horse community?
(g) What are the socio-cultural dimensions and determinants of risk and safety?
(h) What are the socio-cultural barriers and enablers to improve rider safety?
(i) What are the elements of effective behavior change campaigns and programmes that increase rider safety?

## 3. Discussion

In this paper we critically reviewed four dimensions important to horse-related risks to horse riders. Whilst they were presented discretely, they are mutually inclusive within a complex socio-technical network and interspecies interaction that is historically, socially and culturally constructed. In relation to the dimensions of risk, horses, riders and culture, we found that research on risk explains the intricacies of what can go wrong, how often and what the consequences are. Research on horses is also problem focused, arguing why horses are a source of risk. These biases favour risk-management options of acceptance and avoidance. To take full advantage of risk-management strategies of mitigation, further research is required on the extent to and ways in which the behavior of horses can be made more predictable, and riders can be made more capable of predicting their behaviour. There is also a need for research evaluating rider proficiency at performing and self-assessing the physical skills that increase resilience to horse-related injury whilst riding. Finally, we considered the unavoidable and omnipotent cultural context that affects riders’ behaviours, values, attitudes and beliefs regarding risk and safety. Inconsistent levels of voluntary helmet use suggest that increasing the safety of eque-cultures and equestrianism in general will require external legislation and internal transformation. Overall, we found enormous potential for reducing horse-related risk through the risk management strategy of mitigation.

For each element of horse-related risk, we presented a list of research questions. Together, they comprise a multidisciplinary agenda for further research that could significantly reduce accident, injury and death to millions of horse-riders around the world. As these questions are multi-disciplinary, so too do they require data from various sources, including but not limited to:

- Surveys and questionnaires—to obtain a wider perspective on a range of issues, and to generate quantitative results for policy development and advocacy around horse and rider safety [67,68].
- Interviews and focus groups—to explore controversial subjects that pertain to risk and safety of both the horse and rider [45,69].
- Ethnographic research—to study the actual practices of equestrians; and identify risk management strategies consistent with the motivations, beliefs and values of eque-cultures [26,70,71,72,73].
- Media analysis—to identify how particular incidents and risks are reported (or not reported) and relationships to the values, beliefs and practices of equestrians [15,67,74,75].
- Physiometry—to measure rider position and identify physical attributes positively correlated with safety or resistance to being unseated [33,76,77,78].
- Psychometric research into fear [79], risk-taking propensity [80,81] and sensation seeking [82] amongst riders and equestrian discipline—to identify target groups and tailor behavior change interventions [38,83].
- Analysis of accident and injury reports (*i.e*., from inquests, insurance records and hospital admission data)—to enable triangulation of objective and self-report data, especially around risk [6,84,85].
- Inferential modeling - to determine predictors of risk and safety, animal attachment and target group archetyping [86,87].

To ensure that data are translated into effective safety intervention tools that can reduce numbers of horse-related injury and death, researchers should focus on developing initiatives that (a) increase outrage about preventable horse-related injury and death, and (b) reduce horse-related risk, such as:

- Ethical techniques and behavioral interventions to increase the predictability of horses
- Interventions to improve riders’ ability to predict horse behaviour
- Horse safety assessment and decision-making support tools
- Rider safety skills assessment tools
- Validated measure of horse training/riding style
- Behavior change for safe equestrian cultures

## 4. Conclusion: Horse/Human-Related Risk/Safety

As demonstrated throughout this paper, horse-related risk is generated through a complex socio-technical network of risk, horses, humans and culture. Whilst these dimensions have been recognized and in some cases researched, horse-related risk has typically been constructed anthropocentrically; it originates in horses and it impacts humans. Moreover, safety is largely seen as a concern for humans only, despite a desire for safety being a powerful driver of ‘unpredictable’ behavior in horses (and an excellent reward for behavioural interventions). To overcome these biases, there is apparent advantage in the widespread adoption of a more anthrozoological approach to horse-related risk that includes human-related risk as well as human/horse-related safety. With human-horse safety as the ultimate goal, this paper has identified unrealised opportunity to mitigate horse-related risk with behavioural, physical and socio-cultural interventions that could make horses safer mounts, humans safer riders, and equestrianism a safer culture.
